# Extraintestinal Manifestations of Inflammatory Bowel Disease: Unraveling Valvulopathy Connections

**DOI:** 10.7759/cureus.57996

**Published:** 2024-04-10

**Authors:** Akhaled Zaher

**Affiliations:** 1 Internal Medicine, Memorial Sloan Kettering Cancer Center, New York, USA

**Keywords:** aortic regurgitation, crohn disease, ulcerative colitis, mitral regurgitation, inflammatory bowel syndrome

## Abstract

The clinical presentation of inflammatory bowel disease (IBD) includes both gastrointestinal manifestations and extraintestinal manifestations (EIM). Over the past years, a growing number of studies have indicated that patients suffering from IBD have an increased risk of developing cardiovascular disease. Although the precise prevalence of cardiac complications in IBD remains uncertain, emerging evidence suggests a heightened incidence compared to the general population. Valvular heart disease (VHD) in IBD encompasses calcific aortic valve disease, mitral valve prolapses, and endocarditis, potentially associated with chronic inflammation. Considering the role of inflammation in developing cardiovascular manifestations, the management should include preventing flares and maintaining remission for as long as possible. This case highlights the intricate interplay between IBD and cardiovascular complications, particularly valvular abnormalities. We present a 37-year-old male with a history of ulcerative colitis (UC) who was found to have multiple valvular diseases.

## Introduction

Inflammatory bowel disease (IBD) is a term used to describe two chronic conditions: Crohn's disease and ulcerative colitis (UC). These conditions are characterized by chronic inflammation of the gastrointestinal (GI) tract, which can lead to damage in the GI tract [1]. While the primary focus of IBD management has been on the gastrointestinal manifestations, emerging evidence suggests a potential link between IBD and cardiac manifestations. Cardiovascular complications, including pericarditis, myocarditis, venous and arterial thromboembolism, arrhythmias, and conduction disorders, have been reported in IBD patients at a higher frequency than in the general population [2]. The pathogenesis of these cardiac manifestations is believed to be primarily immune-related. The prevalence of aortic regurgitation in IBD is very rare [3]. There have been several case reports in the literature of patients with IBD who developed aortic regurgitation, but its incidence is low [4]. Inflammation in IBD can cause valvopathies, including aortic regurgitation, due to the thickening and shortening of the leaflet. This case highlights the need for cardiac vigilance in IBD management, stressing the importance of integrated care for optimal patient outcomes and preventing severe complications.

## Case presentation

A 37-year-old male with a history of UC diagnosed in 2022 and primary sclerosing cholangitis (PSC) arrived at the emergency department (ED) due to bloody stool and shortness of breath. The bloody bowel movements had begun the day before admission, with over 10 episodes of bright red blood per rectum. These symptoms were accompanied by shortness of breath and lightheadedness that started during his bloody bowel movements.

The patient was diagnosed with UC two years ago, presented with bloody bowel movements, and underwent colonoscopy, revealing pancolitis for which he started taking Mesalamine; however, due to insurance issues, he became non-adherent to therapy and turned to herbal treatment, subsequently achieving remission.

Upon presentation, his vital signs were within normal limits. During the physical examination, he was ill-appearing but not in distress. He had a notable systolic murmur on the cardiac exam, which is a new finding, and a small speck of bright red bleeding was noted in the rectal vault during the rectal examination. He also had pale bilateral conjunctiva and oral mucosa. Laboratory results are summarized (Table 1).

**Table 1:** Laboratory results and reference ranges for key parameters in the case study

Laboratory	Results	Reference range
Hemoglobin	5.7 g/dL	13.2 - 16.6 g/dL
Mean Corpuscular Volume	75 fl	80–100 fl
Alkaline Phosphatase	447 IU/L	44 to 147 IU/L
Gamma Glutamyl Transferase	577 IU/L	8-38 IU/L
Albumin	2.7 g/dL	3.4–5.4 g/dL

**Figure 1:**
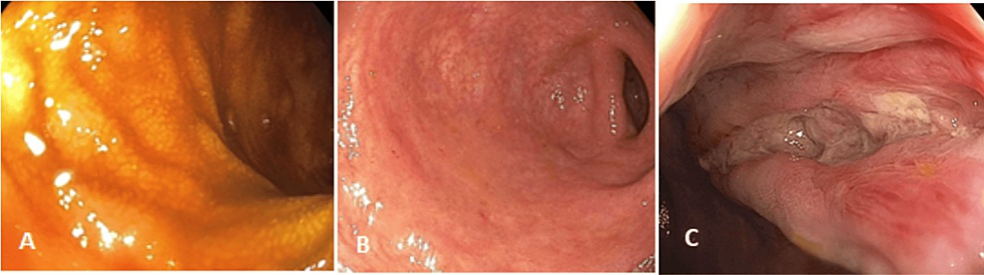
Colonoscopy on the second day of hospitalization. Both the terminal Ileum (A) and the sigmoid (B) had normal mucosal findings. However, a substantial linear nonbleeding ulcer was found in the rectum (C). Absence of inflammation aligns with a Mayo score of 0.

Due to low hemoglobin levels, he received a blood transfusion and was admitted to the medical step-down unit. His infectious workup was negative, including gastrointestinal pathogen panel. His symptoms were attributed to UC flare, and he was started on mesalamine 800 mg PO TID and enema every night. A subsequent colonoscopy on the second day of hospitalization revealed a large linear nonbleeding rectal ulcer (Figure 1) with no signs of inflammation, consistent with a Mayo score of 0.

As the ulcer was in the rectum, enemas were discontinued. Pathology findings were negative. Transthoracic echocardiogram showed severe mitral and aortic regurgitation alongside mild pulmonary and tricuspid regurgitation, which were new findings. His ejection fraction was 45%, with severely dilated left ventricle and atrium. Left ventricular systolic function was low to normal. There was no previous echo available for comparison.

Further evaluation via left heart catheterization showed no apparent coronary artery disease. A subsequent transesophageal echocardiogram revealed severe prolapse and flail of the P2 segment of the posterior mitral leaflet. Consultations with cardiothoracic surgery and cardiology determined that mitral valve repair would be pursued for an outpatient procedure once the patient's condition stabilized. The patient was discharged on mesalamine tablets and metoprolol 25 mg daily for the newly diagnosed mildly reduced ejection fraction and a follow-up with the cardiology service.

## Discussion

The association between IBD and cardiovascular complications, including valvular abnormalities, is an area of growing interest and concern within the medical community. IBD is primarily characterized by chronic inflammation of the gastrointestinal tract. However, the systemic nature of inflammation in IBD can lead to extraintestinal manifestations, affecting various organ systems, including the cardiovascular system. Several studies have documented the link between IBD and an increased risk of cardiovascular complications:

Systemic inflammation and atherosclerosis: Chronic inflammation in IBD is believed to accelerate the process of atherosclerosis, potentially leading to coronary artery disease. Inflammatory cytokines and acute phase reactants can contribute to endothelial dysfunction and plaque formation [5].

Thromboembolic events: Patients with IBD are at a higher risk of thromboembolic events, including deep vein thrombosis and pulmonary embolism, which can indirectly affect cardiovascular health [6].

Valvular abnormalities: Although less common, there have been reports of valvular heart disease in patients with IBD. This includes conditions such as endocarditis and valvulopathy, which may be related to systemic inflammation or the immunosuppressive therapy used in IBD management [7].

The pathophysiology of how IBD affects valves is that excess TNF-α and fibroblastic healing leads to thickening and shortening of the valve leaflets, causing regurgitation. These findings are considered immune-related consequences of IBD [4]. Cardiac involvement in chronic inflammatory disease has already been demonstrated for ankylosing spondylitis or lupus disease. In fact, a systemic increase in cytokines and profibrotic factors reflects upon both heart structure and function. Past studies demonstrated that altered collagen metabolism in IBD could play a role in favoring fibrosis even in organs far from the gut. Mitral valve prolapse and leaflets thickening, diastolic dysfunction, and slightly reduced ejection fraction are possible consequences [8].

Another reason for the patient's valvular abnormalities may be attributed to chronic anemia. When the body lacks an adequate supply of red blood cells, oxygen transport to tissues and organs becomes compromised. This oxygen deprivation can strain the heart, prompting it to work harder to meet the body's oxygen demands. Over time, this can lead to left ventricular hypertrophy, increased LV mass, and LV dilation, potentially leading to deleterious long-term consequences; early studies and meta-analyses have suggested that correcting anemia in patients with heart failure may improve signs, symptoms, left ventricular function, and quality of life [9]. Our patient had an acute on chronic normocytic anemia likely from anemia of chronic disease. The LV function was assessed through echocardiography, revealing potential functional mitral regurgitation and aortic regurgitation, which could be multifactorial; further details on the LV assessment and subsequent follow-up echocardiograms would provide valuable insights into the progression and management of these valvular abnormalities in relation to the patient's anemia.

Early recognition helps prevent complications and minimize the impact on the disease's natural course. Key preventive measures for cardiovascular issues include sustaining remission of IBD for as extended a period as feasible, regular cardiac assessments (comprising physical examinations, blood screenings, electrocardiograms, and transthoracic echocardiography), administering anticoagulants to patients with elevated thromboembolic risks, and addressing both conventional and nonconventional cardiovascular risk factors. In cases of symptomatic severe valvular disease, surgical intervention, such as valve repair or replacement, may be necessary to alleviate symptoms and prevent complications. However, careful consideration of the patient's underlying inflammatory condition and its impact on perioperative risk is essential in treatment decision-making.

## Conclusions

Cardiovascular manifestations in patients with IBD present a heightened incidence compared to the general population, arising from chronic inflammation, medication effects, or genetic factors. Early recognition and preventive measures are paramount to mitigate the impact of disease. Key interventions include maintaining remission, regular cardiologic evaluations, anticoagulation for high thromboembolic risk, and managing cardiovascular risk factors. While the evidence linking IBD to cardiovascular events is robust, prospective studies are needed for further validation. Mechanisms such as inflammation-induced endothelial dysfunction and dyslipidemia are implicated, necessitating focused research.
